# Mutual Metabolic Interactions in Co-cultures of the Intestinal *Anaerostipes rhamnosivorans* With an Acetogen, Methanogen, or Pectin-Degrader Affecting Butyrate Production

**DOI:** 10.3389/fmicb.2019.02449

**Published:** 2019-11-01

**Authors:** Thi Phuong Nam Bui, Henk A. Schols, Melliana Jonathan, Alfons J. M. Stams, Willem M. de Vos, Caroline M. Plugge

**Affiliations:** ^1^Laboratory of Microbiology, Wageningen University & Research, Wageningen, Netherlands; ^2^Laboratory of Food Chemistry, Wageningen University & Research, Wageningen, Netherlands; ^3^Human Microbiome Research Programme, Faculty of Medicine, University of Helsinki, Helsinki, Finland

**Keywords:** butyrate, butyrate-producing bacteria, microbial interaction, gut microbes, *Anaerostipes*

## Abstract

The human intestinal tract harbors diverse and complex microbial communities that have a vast metabolic capacity including the breakdown of complex carbohydrates into short chain fatty acids, acetate, propionate, and butyrate. As butyrate is beneficial for gut health there is much attention on butyrogenic bacteria and their role in the colonic anaerobic food chain. However, our understanding how production of butyrate by gut microorganisms is controlled by interactions between different species and environmental nutrient availability is very limited. To address this, we set up experimental *in vitro* co-culture systems to study the metabolic interactions of *Anaerostipes rhamnosivorans*, a butyrate producer with each of its partners; *Blautia hydrogenotrophica*, an acetogen; *Methanobrevibacter smithii*, a methanogen and *Bacteroides thetaiotaomicron*, a versatile degrader of plant cell wall pectins; through corresponding specific cross-feeding. In all co-cultures, *A. rhamnosivorans* was able to benefit from its partner for enhanced butyrate formation compared to monocultures. Interspecies transfer of hydrogen or formate from *A. rhamnosivorans* to the acetogen *B. hydrogenotrophica* and in turn of acetate from the acetogen to the butyrogen were essential for butyrate formation. *A. rhamnosivorans* grown on glucose supported growth of *M. smithii* via interspecies formate/hydrogen transfer enhancing butyrate formation. In the co-culture with pectin, lactate was released by *B. thetaiotaomicron* which was concomitantly used by *A. rhamnosivorans* for the production of butyrate. Our findings indicate enhanced butyrate formation through microbe-microbe interactions between *A. rhamnosivorans* and an acetogen, a methanogen or a pectin-degrader. Such microbial interactions enhancing butyrate formation may be beneficial for colonic health.

## Introduction

The human large intestine is an anoxic ecosystem with microbial communities that are highly dynamic and regulated by a variety of host and environmental factors, including nutrition (Zoetendal and de Vos, 2014). Nevertheless, each individual carries a personalized gut microbiome and multiple studies revealed high-level structures, termed enterotypes, with similarities in composition (Arumugam et al., 2011). Three enterotypes have been described with *Bacteroides, Prevotella*, and *Ruminococcus* as their main drivers. Positive or negative co-occurrence of different taxonomic groups with these three main drivers have been proposed, based on statistical analyses, that could be used to predict species interactions at functional level. However, such microbial network predictions should be tested and therefore it is essential to study the microbial interspecies interactions *in vitro* under physiologically relevant conditions that mimic the human intestinal tract.

Fermentation of carbohydrates in the colon produces a variety of bioactive compounds, especially short chain fatty acids (SCFAs) such as butyrate which is crucial to maintain a healthy gut as it fuels enterocytes, signals to the host and is a critical mediator of the colonic inflammatory response (Zimmerman et al., 2012). Butyrate-producing bacteria, one of the core functional groups in the human intestine, are reduced in cell number in a variety of diseases, including inflammatory bowel disease (IBD) and diabetes (Clemente et al., 2012). Therefore, there is great interest in understanding and defining microbial interactions that involve butyrogenic bacteria and influence butyrate production.

Lactate and hydrogen are also important fermentation products of carbohydrate degradation (Flint et al., 2012). Accumulation of lactate or hydrogen in the gut environment results in gastrointestinal disorders (Hove et al., 1994). In addition, the accumulation of hydrogen is known to hinder re-oxidation of NADH, leading to a decrease in ATP yield and SCFA production (Rey et al., 2010). As a consequence it may affect the host energy balance via reduced SCFA turnover. However, lactate and hydrogen can be used efficiently by different groups of bacteria in the gut. Lactate can be used by butyrate-producing bacteria in the presence of acetate with the ratio of lactate:actate:butyrate as 2:1:1.5 using the acetyl-CoA pathway (converting acetyl-CoA to butyrate) (Duncan et al., 2004). Known bacteria that convert lactate and acetate into butyrate mostly belong to *Clostridium* cluster XIVa, including *Anaerostipes* spp. and *Eubacterium hallii*. Other intestinal butyrate producers convert glucose plus acetate, but not lactate plus acetate, such as *Faecalibacterium prausnitzii*, which belongs to *Clostridium* cluster IV and *Roseburia* spp. (Louis and Flint, 2009). Isotope labeling studies have identified lactate and acetate as important precursors of butyrate production in mixed communities from the gut (Bourriaud et al., 2005; Muñoz-Tamayo et al., 2011). It has been shown that some strains of bifidobacteria and lactobacilli have lactate- and acetate-based cross-feeding interaction with butyrate-producing bacteria in inulin-type fructans (Moens et al., 2017). Similar cross-feedings have been reported between infant bifidobacteria with *E. hallii* on mucin and L-fucose/fucosyllactose (Duncan et al., 2004; Samuel and Gordon, 2006; Coenen et al., 2007; Bunesova et al., 2017; Schwab et al., 2017). Methanogens and acetogens are important functional groups in the gut that are responsible for hydrogen removal through the production of methane and acetate, respectively (Nakamura et al., 2010; Smith et al., 2018). While the prevalence of methanogenesis in the gut is varying in different geographic and ethnic populations, ranging from 34 to 87% (Levitt et al., 2006), acetogenesis is a dominant and consistently represented metabolic process in the human colon (Rey et al., 2010; Smith et al., 2018). *Methanobrevibacter smithii* is a major intestinal methanogenic archaeon, which is able to grow on H_2_/CO_2_ or formate (Eckburg et al., 2005; Abell et al., 2006; Scanlan et al., 2008; Nkamga et al., 2015). *Blautia hydrogenotrophica* is a dominant intestinal acetogen which is able to grow not only on H_2_/CO_2_ employing the Wood–Ljungdahl pathway but also ferments carbohydrates (Bernalier et al., 1996). Both the methanogen and acetogen have shown to efficiently remove hydrogen in co-culture experiments on cellulose and xylan (Robert et al., 2001; Chassard and Bernalier-Donadille, 2006) where *M. smithii* also consumed formate in a germ-free mouse model (Samuel and Gordon, 2006). It has been shown that *Methanobacterium ruminantium* influenced glucose fermentation by *Selenomonas ruminantium*, leading to a noticeable decrease in lactate and propionate production (Chen and Wolin, 1977). Another co-culture study using *in vivo* gnotobiotic mice and *in vitro Bacteroides thetaiotaomicron* and *B. hydrogenotrophica* showed that *B. hydrogenotrophica* was able to utilize hydrogen produced by *B. thetaiotaomicron* via the Wood–Ljungdahl pathway which in turn allowed *B. thetaiotaomicron* to regenerate NAD^+^ to be used for glycolysis (Rey et al., 2010).

The human microbiome composition and its activities are significantly influenced by the carbohydrate content of the dietary intake (Walker et al., 2011; O’Keefe et al., 2015; Hansen and Sams, 2018). Therefore, understanding trophic chains utilizing food-related carbohydrates and resulting in butyrate formation would contribute to mechanistic insight. Pectin is one of the high molecular weight polymeric carbohydrates and abundant in fruits and vegetables and frequently applied as gelling agent in food (Samuel et al., 2006). Pectin is a complex polysaccharide with mainly galacturonic acid (GalA) in its backbone, either present in long GalA sequences in homogalacturonan structural elements or the highly substituted rhamnogalacturonan I segments. The rhamnogalacturonan I backbone consists of dimers of rhamnose- GalA which galactose and arabinose rich side chains are connected to O-4 position of rhamnose (Voragen et al., 2009). Pectin has been shown to have the potential to modulate gut microbiota in a positive way by targeting beneficial bacteria to enable more balanced microbiomes (Larsen et al., 2019). Members of the genus *Bacteroides* have the capacity to metabolize a large variety of plant-derived glycans including pectin (Salyers et al., 1977; Jensen and Canale-Parola, 1986; Lammerts van Bueren et al., 2017) with *B. thetaiotaomicron* as one of the prominent glycan degraders in the gut (Dongowski et al., 2000). The human intestinal species *B. thetaiotaomicron* encodes a huge repertoire of carbohydrate degrading activities, including malto-oligosaccharides and starch and has the ability to switch between diet- and host-derived carbohydrates (Reeves et al., 1996). Recently, new enzyme families have been discovered from *B. thetaiotaomicron* that cleave off RG-II, a highly complex glycan (Ndeh et al., 2017) and one of these glycosidase hydrolases has been further characterized (Labourel et al., 2019). However, it has not been studied how *B. thetaiotaomicron* interacts with intestinal microbes including butyrate-producing bacteria when growing on pectins, though a substantial amount of these polymers are present in daily food.

*Anaerostipes rhamnosivorans* is a butyrate-producing bacterium, isolated from infant stool (Bui et al., 2014). The genus *Anaerostipes* is among the top fifteen abundant genera in the human intestinal microbiome (Arumugam et al., 2011). *A. rhamnosivorans* is able to perform mixed acid fermentations with many sugars, including rhamnose for its butyrogenesis. So far, butyrate production from rhamnose is a new feature for intestinal microbes. *A. rhamnosivorans* produces butyrate from sugars but also from lactate plus acetate.

Altogether, we carefully selected the community members being abundant and typical members of the human gut microbiome, and involved in butyrate production. Here, we present metabolic interactions between *A. rhamnosivorans* and a number of important gut microorganisms *B. hydrogenotrophica* (former *Ruminococcus hydrogenotrophicus*), an acetogen isolated from human stool (Liu et al., 2008), *B. thetaiotaomicron*, a degrader of various pectin substrates (Dongowski et al., 2000) and *M. smithii*, the most abundant intestinal methanogen (Gaci et al., 2014). The results show enhanced butyrate formation through microbe-microbe interactions, between *A. rhamnosivorans* and the acetogen, methanogen or pectin-degrader.

## Materials and Methods

### Growth of the Microorganisms

*Anaerostipes rhamnosivorans* (DSM 26241^*T*^) was isolated from infant stool (Bui et al., 2014), *B. thetaiotaomicron* (DSM 2079^*T*^), *B. hydrogenotrophica* (DSM 10507^*T*^), and *M. smithii* (DSM 861^*T*^) were obtained from the German Collection of Microorganisms and Cell Cultures (DSMZ, Brunswick, Germany). The metabolism of all microbes is presented in Table 1.

**TABLE 1:** Microorganisms used in this study and metabolic profiles from relevant carbon sources.

**Strain**	**Substrate**	**Metabolite**	**References**	**Origin**
*Anaerostipes Rhamnosivorans* DSM 26241^T^	Glucose	Butyrate, lactate, formate, acetate, hydrogen	Bui et al. (2014)	Human intestine
	Rhamnose	1,2-propanediol, butyrate, lactate, formate, acetate, hydrogen		
	Lactate/acetate	Butyrate, hydrogen		
*Blautia hydrogenotrophica* DSM 10507^T^	H_2_/CO_2_	Acetate	Bernalier et al. (1996)	Human intestine
	Glucose	Lactate, acetate	Liu et al. (2008)	
*Bacteroides thetaiotaomicron* DSM 2079^T^	Glucose	Succinate, propionate, acetate	Pan and Imlay (2001)	Human intestine
	Rhamnose	1,2-propanediol	Patel et al. (2008)	
	Pectins	Acetate, propionate	Dongowski et al. (2000)	
*Methanobrevibacter smithii* DSM 861^T^	H_2_/CO_2_ or formate	CH_4_	Miller et al. (1982)	Human intestine

*The information of the microorganisms used was collected and summarized from Miller et al. (1982), Bernalier et al. (1996), Dongowski et al. (2000), Pan and Imlay (2001), Liu et al. (2008), Patel et al. (2008), and Bui et al. (2014).*

All microorganisms were routinely maintained under anaerobic conditions in 120-ml serum bottles. Reinforced Clostridial Medium (RCM, DIFCO) was used to pregrown *A. rhamnosivorans*, *B. thetaiotaomicron*, and *B. hydrogenotrophica*. *M. smithii* was grown in bicarbonate-buffered anaerobic media (Stams et al., 1993; van Gelder et al., 2012) supplemented with 0.2 g/l yeast extract; 0.05 g/l cystein; 2 mM acetate as carbon source and H_2_/CO_2_ (80:20, v/v) at a pressure of 1.7 bar as energy source. All co-culture experiments were performed in serum bottles sealed with butyl-rubber stoppers containing bicarbonate buffered anaerobic media with certain substrates as carbon and energy sources at 37°C under a gas phase of 1.7 atm of N_2_/CO_2_ (80:20, v/v). In addition, vitamins and sodium sulfide (1 mM) were added to the medium from sterile stock solutions as growth factor and reducing agent, respectively (Stams et al., 1993). All stock solutions were prepared, sterilized and kept in serum bottles with a headspace of N_2_.

### Co-culture of *A. rhamnosivorans* and *B. hydrogenotrophica* in Lactate

The co-culture experiments were performed in 50 ml bicarbonate buffer medium supplemented with 2 g/l yeast extract, 2 g/l tryptone to support the growth of *B. hydrogenotrophica*. Lactate was added from a 1 M sterile anaerobic stock solution to a concentration of 20 mM in the co-culture as the carbon and energy source. Equal amounts of the overnight precultures of each microorganism (2%, v/v) were inoculated simultaneously into the medium to complete the co-cultures. Monocultures of *A. rhamnosivorans* and *B. hydrogenotrophica* in the bicarbonate buffered media supplemented with 2 g/l yeast extract, 2 g/l tryptone, containing lactate were used as controls. Gas and liquid samples were collected over time for H_2_ and organic acid analysis, respectively.

### Co-culture of *A. rhamnosivorans* and *M. smithii* in Glucose

*Methanobrevibacter smithii* and *A. rhamnosivorans* were pre-cultured in the media as described above. *M. smithii* was incubated for a week with H_2_/CO_2_ (80:20, v/v) before the headspace was changed to N_2_/CO_2_ (80:20, v/v). The co-culture was established by adding 1 ml (2% v/v) of an overnight culture of *A. rhamnosivorans* into the 1-week pregrown culture of *M. smithii* in 120-ml serum bottles with 50 ml bicarbonate buffered medium. Glucose from 1 M sterile stock solution was added to the co-culture to a concentration of 20 mM. Monocultures of *A. rhamnosivorans* in the same bicarbonate buffered medium containing 20 mM glucose as substrate were used as control. In addition, for the *M. smithii* control, a 1 week pregrown culture was supplemented with 20 mM glucose and the head space was changed N_2_/CO_2_ (80:20, v/v). Gas and liquid samples were collected over time for H_2_, CH_4_, and organic acid analysis, respectively.

### Co-cultures of *A. rhamnosivorans* and *B. thetaiotaomicron* in Pectins

All co-culture experiments were performed using an anaerobic, 10 ml bicarbonate-buffered mineral medium (Stams et al., 1993). Pectins were prepared in concentrated stock solutions (50 g/l SBPOS; 10 g/l RG-I apple; 50 g/l SBP6230; 100 g/l SBP; 20 g/l RG-I potato; 50 g/l SSPS) in anaerobic serum bottles and sterilized before adding into the medium. See Table 2 for full names of the pectins. The overnight cultures of *A. rhamnosivorans* and *B. thetaiotaomicron* were used as inocula. Two series of co-culture experiments were carried out, with different amounts of pectins.

**TABLE 2:** Dietary pectin fractions and their quantities in the first series of co-cultures of *A. rhamnosivorans* and *B. thetaiotaomicron*.

**Substrate**	**Abbreviation**	**Quantity per bottle (g/l)**	**References**
Sugar beet pectin	SBP	2	Remoroza et al. (2012)
Soy pectin	SSPS	1	Coenen et al. (2007)

In the first co-culture experiment, 0.4 ml of substrates was added into 9 ml bicarbonate-buffered mineral medium to make the indicated quantities (Concentrations of respective pectins see Table 2). Finally, 2% (v/v) of the overnight precultures of each microorganism were added. In parallel, *A. rhamnosivorans* and *B. thetaiotaomicron* were grown in monocultures in the bicarbonate buffered medium containing SBP, SBP6230, SBPOS, RG-I potato, and RG-I apple and SSPS as substrates for the comparisons. Gas and liquid samples were collected after 8 days of incubation for H_2_ and organic acid analysis, respectively.

The second co-culture experiment was performed in bicarbonate buffered medium with increased amounts of SBP (4.68 g/l) and SSPS (5.49 g/l), and a different ratio of the two bacteria that were inoculated: 8% (v/v) of *A. rhamnosivorans* and 2% (v/v) of *B. thetaiotaomicron*. Monocultures of *A. rhamnosivorans* and *B. thetaiotaomicron* in the same conditions were used for comparison. Gas and liquid samples were collected after 6 days of incubation for H_2_ and organic acid analysis. All experiments were performed in duplicate.

### Analytical Methods

Glucose, organic acids, and alcohols were measured on a Thermo Scientific Spectra HPLC system equipped with a Agilent Metacarb 67 H 300 × 6.5 mm column kept at 45°C and running with 0.005 M H_2_SO_4_ as eluent. The detector was a refractive index detector. The eluent flow was 0.8 ml/min. All analyses were performed in duplicate. H_2_ and CH_4_ measurement were performed by a Shimadzu GC-14B gas chromatography (Shimadzu, Kyoto, Japan) equipped with a Molsieve 13X column (Varian, Middelburg, Netherlands) and a thermal conductivity detector. Injection volume was 0.2 ml and detector temperature was 150°C. The carrier gas was argon at the flow rate of 30 ml/min. All analyses were performed in duplicate.

### Enumeration of Bacteria in Co-cultures by Microscopic Counting

Due to their distinct morphologies, the relative abundance of each microbe in the co-cultures could be determined by microscopic counting using a Leica DM 2000 phase contrast microscope at 1000× magnification. Bacterial cultures were sampled during the exponential phase. Each microscopic slide was covered by a thin layer of soft agar suspension (1% w/v Noble agar, DIFCO). Subsequently, 2 μl cultures were placed on the prepared slides and air-dried for 5 min before being observed. Images were captured by using a Leica DMC2900 camera (Leica Microsystem, Germany). At least 50 microscopic fields were counted for each co-culture. The values presented represent the average of all counted fields.

## Results

### Co-culture of *A. rhamnosivorans* and *B. hydrogenotrophica* in Lactate

**FIGURE 1:**
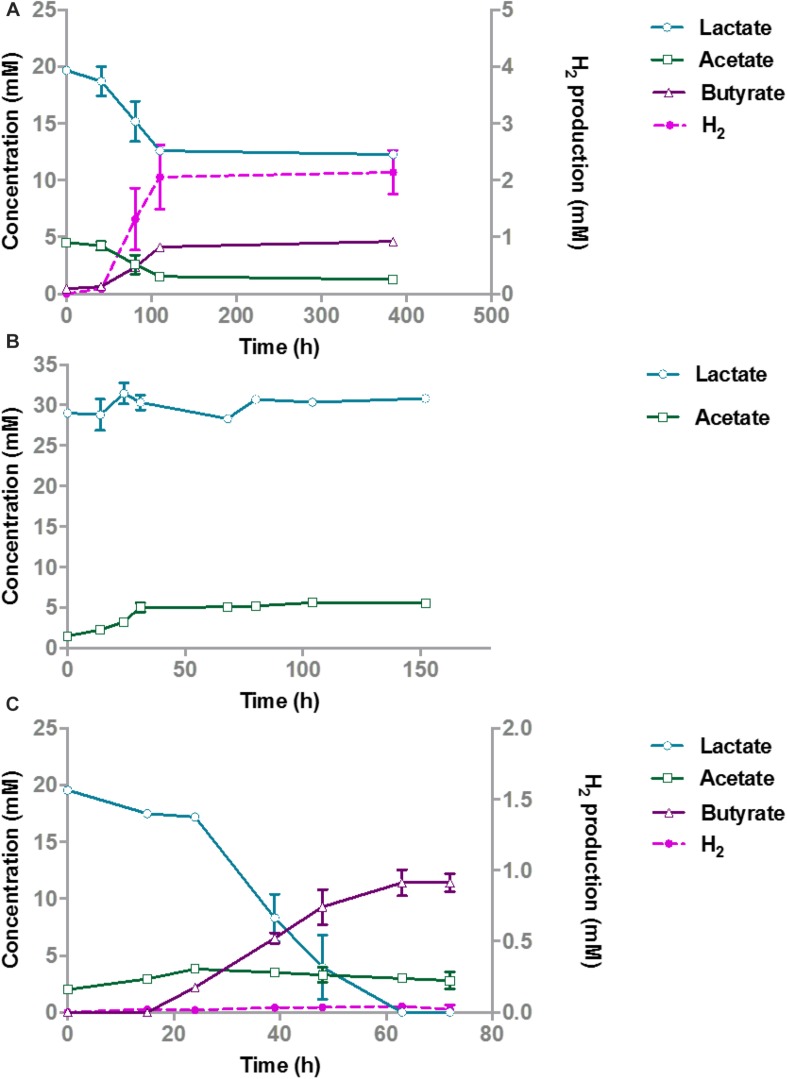
Lactate degradation and product formation in monocultures of *A. rhamnosivorans* **(A)**, *B. hydrogenotrophica* **(B)** and co-cultures of *A. rhamnosivorans* and *B. hydrogenotrophica* **(C)**. Lactate, acetate and butyrate concentrations are shown on the left *y*-axis (mM), H_2_ is plotted on the right *y*-axis (mM). All experiments were performed in duplicates and mean values were shown here.

Butyrate-producing bacteria are able to convert 2 moles of lactate and 1 mole of acetate to form 1.5 mole of butyrate such as *E. hallii* and *Anaerostipes caccae* (Duncan et al., 2004). *A. rhamnosivorans* indeed converts lactate and acetate to butyrate and hydrogen (Bui et al., 2014) while *B. hydrogenotrophica* has been reported to convert H_2_/CO_2_ to acetate (Bernalier et al., 1996). To investigate the cross-feeding in the co-culture between the butyrate-producing bacterium and the acetogen in presence of a small quantity of acetate, 20 mM lactate and 5 mM acetate were fed as substrates to mono- and co-culture. In the monoculture, *A. rhamnosivorans* metabolized lactate plus acetate (with acetate being transferred from the pre-culture with inoculation) to butyrate, but lactate consumption and butyrate formation stopped when acetate was depleted (Figure 1A). In pure culture, *B. hydrogenotrophica* produced a small amount of acetate during the first 24 h, likely from utilization of components in yeast extract or peptone, but acetate production stopped during further incubation (Figure 1B). Thus, neither of the microbes grew on only lactate in monoculture.

After 72 h, the OD600 In the co-cultures increased from 0.07 to 0.4 and 20 mM lactate was completely converted to 11.4 mM butyrate (Figure 1C). Hydrogen was formed as a minor product (<0.05 mM) during the co-culture experiment and acetate increased from 2 to 3.8 mM in 24 h and slowly decreased afterward to 2.8 mM after 72 h. Hydrogen and acetate levels were low showing the balance between the production and utilization of these two compounds by *A. rhamnosivorans* and *B. hydrogenotrophica*. The carbon and electron balances are presented in Supplementary Table S1. The calculated electron recovery was close to 100%. No loss of carbon was observed, assuming that CO_2_ formed was equal to the amount of butyrate that was produced, indicating all major products were detected. An equal ratio of *A. rhamnosivorans* and *B. hydrogenotrophica* after 72 h was observed using the microscopic counting method, confirming the comparable activities of these two organisms in this co-culture (Supplementary Data S1 and Figure 1).

### Co-culture of *A. rhamnosivorans* and *M. smithii* in Glucose

**FIGURE 2:**
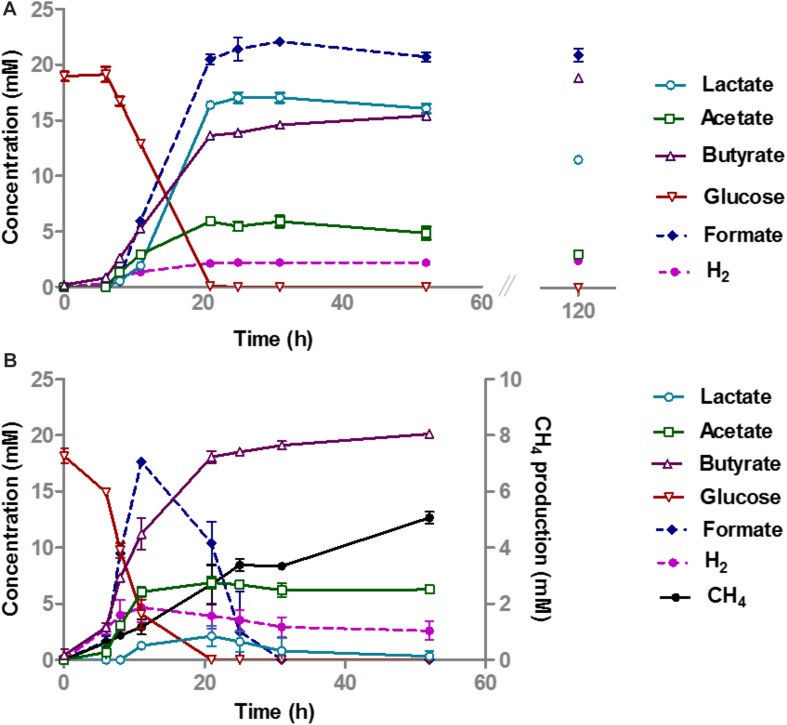
Glucose conversion and product formation in monocultures of *A. rhamnosivorans* **(A)** and co-cultures of *A. rhamnosivorans* plus *M. smithii* **(B)**. **(B)** Secondary *y*-axis indicates the production of CH_4_ and H_2_ in mM. All experiments were done in duplicates. Shown values are means of duplicates.

A monoculture of *A. rhamnosivorans* converted 20 mM glucose to butyrate, lactate, formate, acetate, and hydrogen (Figure 2A), while there was no growth of the pure culture of *M. smithii* in medium with 20 mM glucose (data not shown). After the rapid depletion of glucose within 24 h, a metabolic switch was needed to start metabolizing the lactate plus acetate that was formed. *A. rhamnosivorans* slowly converted 1 mM lactate and 0.6 mM acetate to butyrate in the next 38 h. This conversion was ongoing during 5-day incubation, producing additional 3.5 mM butyrate as compared to 52 h (Figure 2A).

In the co-culture of *A. rhamnosivorans* and *M. smithii*, glucose fermentation and methane production occurred simultaneously (Figure 2B). The OD600 of the co-cultures, increased from 0.28 to 2.3, similar to the monoculture of *A. rhamnosivorans*. Glucose was consumed faster during the first 12 h as compared to the monoculture (Figure 2) and was completely depleted after 21 h in both the monoculture and co-culture. In the co-culture, only a small amount of lactate was detected after 21 h (2.1 mM) and this was completely converted to butyrate after 48 h. In contrast, lactate production was much higher in the monoculture (16.8 mM). After 52 h incubation, 19.5 mM butyrate was formed in the co-culture, which was higher than that of the monoculture of *A. rhamnosivorans* (15.1 mM). The net acetate production was always slightly higher in the co-culture (6.3 mM) than in the monoculture (4.7 mM) of *A. rhamnosivorans*. In the co-culture, formate and H_2_ increased within 12 h and thereafter were rapidly converted to CH_4_ indicating growth of the methanogen. 5.1 mM CH_4_ was formed at the end of growth in the co-culture. The carbon and electron balances are presented in Supplementary Table S2. CO_2_ was calculated based on theoretical stoichiometry of glucose fermentation (Supplementary Table S2). The calculated carbon recoveries were 102 and 118% of which butyrate was accounted for 71 and 43% in co-culture and monoculture, respectively. Cell numbers of *A. rhamnosivorans* were 2.6 times higher than those of *M. smithii* (Supplementary Data S2). Our data indicates that the growth of methanogenic partner was clearly dependent on the butyrogenic partner, and in turn the methanogen influenced the metabolism of its partner. As the methanogen was not able to consume anything else than formate or H_2_/CO_2_, the growth of this particular microbe had to be dependent on those that produce these metabolites. It is known that many butyrate producing bacteria produce formate and/or H_2_/CO_2_ as products of fermentation, hence, interaction with butyrate-producing bacteria might be one of key modes for the methanogen to obtain substrates from the gut bacteria in the colon.

### Co-culture of *A. rhamnosivorans* and *B. thetaiotaomicron* in Pectins

Co-cultures of *A. rhamnosivorans and B. thetaiotaomicron* were set up and the first experiments showed that different pectin fractions were degraded and that some butyrate was formed from the degradation of pectins SBP and SSPS but not from SBP6230, SBPOS, RG-I potato, and RG-I apple (Supplementary Table S3). This indicated that there was an interaction between *B. thetaiotaomicron* and *A. rhamnosivorans* in SBP and SSPS. Although all pectins contain the same sugar building blocks linked through the same linkages, the amount and sequence of the different sugar building blocks vary amongst the pectins (Voragen et al., 2009). SBP and SSPS are rich in GalA, rhamnose, arabinose, and galactose (Sengkhamparn et al., 2009). *B. thetaiotaomicron* showed the capabilities of fermenting the two pectins SBP and SSPS with acetate as the major end product while *A. rhamnosivorans* was not able to convert any of the pectins in monoculture (Supplementary Table S3). Notably, the amount of SBP was two times higher than that of SSPS, leading to a larger amount of acetate production from SBP comparing to SSPS in both monocultures and co-cultures.

To further investigate the interaction between *B. thetaiotaomicron* and *A. rhamnosivorans* in SBP and SSPS, the co-culture experiments were repeated using higher amounts of these two pectins with similar quantity (5.49 g/l of SSPS and 4.68 g/l SBP). The inoculum ratio was 4: 1 (v/v) of pregrown cells of *A. rhamnosivorans* and *B. thetaiotaomicron*. The two bacteria were pregrown in RCM medium overnight reaching OD600 of 2 before being used as inocula. After 6 days of incubation, the co-cultures reached an OD600 of 1 and 1.6 with SBP and SSPS, respectively. The two pectins SBP and SSPS have a different monosaccharide composition (Supplementary Table 1 and Supplementary Table S4) that resulted in growth up to different cell densities and product formation (Supplementary Table S3). After 6 days, the co-culture consumed 10% more SBP than the monoculture with GalA being the most utilized monosaccharide. In contrast, the consumption of SSPS in the co-culture was 7% less than that in monoculture where the abundantly present sugars arabinose and galactose were utilized more readily than the other monosaccharides (Supplementary Table S4). Acetate production with SSPS was higher than with SBP, in the monocultures as well as the co-cultures. Altogether, in monoculture *B. thetaiotaomicron* appeared to metabolize better with SSPS as compared to SBP when similar amounts of substrates were provided. This was not seen in the Supplementary Table S3 due to the difference in the initial quantity of SSPS and SBP in the culture in which SBP was added twice as much as SSPS. Interestingly, in co-cultures butyrate production from SBP was higher than that of SSPS (5 mM and 1.8 mM with SBP and SSPS, respectively). This could be counted to the elevated amount of lactate formation in SBP in comparison to SSPS. Trace amounts of hydrogen were detected in the co-cultures, but not in the monocultures (1.2 mM with SBP and 0.5 mM with SSPS; Table 3). The fact that no lactate could be detected with butyrate formation in co-cultures suggests the almost complete conversion of lactate formed by *B. thetaiotaomicron* into butyrate by *A. rhamnosivorans*. Ethanol and propionate production by *B. thetaiotaomicron* was slightly reduced while the acetate concentration stayed almost the same in the co-cultures, suggesting that the presence of *A. rhamnosivorans* might have influenced the metabolism of its partner. In addition, no 1,2-propanediol, the end product of rhamnose fermentation by *A. rhamnosivorans*, was detected in either the co-cultures or monocultures that was in a good agreement with only little amounts of rhamnose content fermented (Table 1 and Supplementary Table S4). Cell counts showed a 1:10 ratio of *A. rhamnosivorans* and *B. thetaiotaomicron* after growth with both substrates while the inoculum ratio was 4: 1 showing that mainly *B. thetaiotaomicron* was growing (Supplementary Data S3A,B).

**TABLE 3:** Product formation from monocultures of *B. thetaiotaomicron* and co-cultures of *B.thetaiotaomicron* and *A. rhamnosivorans* with SBP and SSPS.

**Substrate**	**Strain(s)**	**Time (days)**	**Lactate (mM)**	**Acetate (mM)**	**Succinate (mM)**	**Propionate (mM)**	**Ethanol (mM)**	**Butyrate (mM)**	**Hydrogen (mM)**
SBP	*B. thetaiotaomicron*	*T* = 0	ND	3.25 ± 1.39	ND	ND	ND	ND	ND
		*T* = 6 days	3.56 ± 0.36	25.5 ± 2.4	0.19 ± 0.05	2.09 ± 0.13	1.54 ± 0.27	ND	ND
	*B. thetaiotaomicron* + *A. rhamnosivorans*	*T* = 0	ND	5.63 ± 0.14	0.24 ± 0.17	ND	ND	1.85 ± 0.1	ND
		*T* = 6 days	ND	27.96 ± 0.67	0.4 ± 0.1	ND	1.04 ± 0.1	6.83 ± 0.27	1.19
SSPS	*B. thetaiotaomicron*	*T* = 0	0.37 ± 0.04	1.8 ± 0.2	ND	ND	ND	ND	ND
		*T* = 6 days	1.06 ± 0.16	38.25 ± 1.28	0.12 ± 0.04	0.96 ± 0.02	4.55 ± 0.33	ND	ND
	*B. thetaiotaomicron* + *A. rhamnosivorans*	*T* = 0	0.37 ± 0.02	3.25 ± 0.3	0.04 ± 0.05	ND	ND	1.7 ± 0.15	ND
		*T* = 6 days	0.05 ± 0.06	38.45 ± 0.31	0.19 ± 0.02	0.69 ± 0.35	3.33 ± 0.002	3.54 ± 0.2	0.53 ± 0.054

*Product formation was determined after 6 days of growth. No growth was observed in monoculture of *A. rhamnosivorans* in SBP or SSPS. Mean and data range are shown. ND, not detected. All experiments were done in duplicate (mM); 5.49 g/l of SSPS and 4.68 g/l SBP was added as substrate.*

## Discussion

Bacteria belonging to *Clostridium* cluster XIVa contribute to intestinal butyrate formation (Louis et al., 2007). Several butyrate-producing bacteria have secondary interactions with hydrogen scavenging microorganisms (Duncan et al., 2004; Chassard and Bernalier-Donadille, 2006). Many butyrate-producing bacteria are capable of performing a mixed acid fermentation with monomeric sugars and only a few of these butyrogens can utilize dietary complex carbohydrates (Chassard and Bernalier-Donadille, 2006; Lopez-Siles et al., 2012; Scott et al., 2014). Most of them rely on primary degraders that are able to utilize indigestible dietary fractions in the large intestine (Belenguer et al., 2006). The results presented here provide information on interactions of butyrate-producing bacteria with other intestinal microbes on glucose, lactate and the dietary pectin fractions.

**FIGURE 3:**
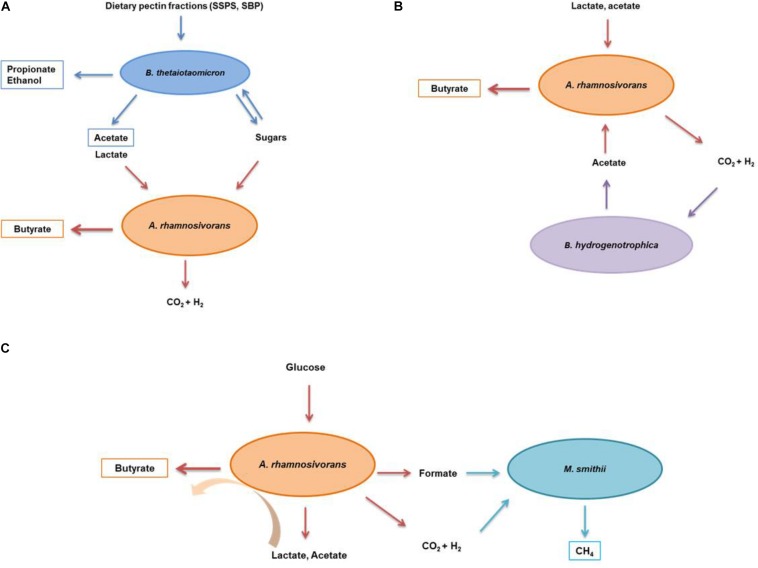
Schematic illustration of the interactions between *A. rhamnosivorans* and intestinal microbes in different substrates. The proposed metabolic interaction between *Anaerostipes rhamnosivorans* and *Bacteriodes thetaiotaomicron* in dietary pectin fractions **(A)**; and *Blautia hydrogenotrophica* in lactate and acetate **(B)**; and *Methanobrevibacter smithii* in glucose **(C)**. Orange, blue, purple, and indigo arrows indicate the conversion by *Anaerostipes rhamnosivorans, Bacteriodes thetaiotaomicron, Blautia hydrogenotrophica*, and *Methanobrevibacter smithii*, respectively. End products of the co-cultures and monocultures are in rectangle panels while substrates and intermediates are not.

Understanding how the production of butyrate by gut microorganisms is controlled by interactions with neighboring species is an important open challenge in microbiome research. In such a complex microbial community as the human intestinal microbiome, a simplified co-culture study is fundamental to understand potential microbe-microbe interactions. With special focus on the butyrate-producing bacterium *A. rhamnosivorans* we showed for the first time its enhanced butyrate formation as a result of the syntrophy with the different partner microorganisms. Intestinal microbial mixtures mostly convert lactate into butyrate (Bourriaud et al., 2005), though detailed microbial community analysis to identify the main lactate degraders in the mixed community is missing. Lactate is produced by many intestinal microbes *in vitro*, however, it is not detected or at very low level in the human intestine due to its rapid conversion (Bourriaud et al., 2005). Lactate accumulation is undesired, as it may result in gastrointestinal disorders (Hove et al., 1994). *E. hallii* is able to convert lactate and acetate, that are produced by *Bifidobacterium adolescentis*, into butyrate (Belenguer et al., 2006). In our co-culture study, it was clearly shown that lactate was metabolized via efficient cross-feeding between *A. rhamnosivorans* and *B. hydrogenotrophica* by means of interspecies transfer of hydrogen and acetate; a bidirectional metabolic cross feeding (Figure 1). Hydrogen transfer between xylanolytic or cellulolytic bacteria and *B. hydrogenotrophica* has been shown previously (Robert et al., 2001; Chassard and Bernalier-Donadille, 2006). A recent study also showed that *B. hydrogenotrophica* was able to remove hydrogen when co-cultured with *B. thetaiotaomicron* (Rey et al., 2010). In our study, it is clear that the cross-feeding between *A. rhamnosivorans* and *B. hydrogenotrophica* resulted in simultaneous growth of both microorganisms. H_2_/CO_2_ conversion (produced by *A. rhamnosivorans*) by the acetogenic *B. hydrogenotrophica* resulted in the formation of acetate, which is used together with lactate by the butyrate-producing bacterium. *A. rhamnosivorans* required a small amount of external acetate to start lactate conversion to butyrate. These two reactions are depicted in Figure 3. This cross-feeding between two dominant species in the human intestine may contribute to (i) maintain a lactate balance in the gut, (ii) prevent lactate and hydrogen accumulation, and (iii) convert non-beneficial compounds (H_2_, CO_2_) to a beneficial butyrate in the gut.

More than 50% of humans harbor methanogenic archaea in their gut where these are likely to form syntrophic relationships with anaerobic bacteria (Chaudhary et al., 2015). In present study, we observed a significant impact of the methanogen *M. smithii* on the glucose metabolism of the butyrogenic *A. rhamnosivorans*. The activity of the methanogen in the co-culture led to a decrease in lactate, and an increase in butyrate production, compared to the monocultures. The methanogen consumed the hydrogen and formate produced during glucose fermentation by the butyrogenic partner (Figure 2). This resulted in a reduction of lactate formation. As a consequence, more butyrate was formed from glucose by *A. rhamnosivorans* as compared to a pure culture of *A. rhamnosivorans*. H_2_ and formate were effectively transferred between the butyrogenic partner and the methanogen, and growth of the methanogen was dependent on the butyrogenic bacterium. As a result, no formate and only trace amounts of H_2_ were detected in the co-culture. It has been previously shown that growth of *M. smithii* enhanced the efficiency of dietary glycan-fermentation in the animal gut by removal of hydrogen and formate (Samuel and Gordon, 2006). Another study on co-occurrence of *M. smithii* and bacteria showed that several species of *Clostridium* cluster IV and XIVa were positively associated with *M. smithii* indicating a syntrophic relationship (Hansen et al., 2011). In previous studies using defined co-cultures *M. smithii* was capable of utilizing hydrogen produced by *Roseburia intestinalis* when co-cultured on xylan with no additional butyrate production (Chassard and Bernalier-Donadille, 2006) or by fibrolytic strains when paired on cellulose without enhancement of cellulose degradation (Robert et al., 2001). Our co-culture study between *A. rhamnosivorans*, belonging to *Clostridium* cluster XIVa, and *M. smithii* on glucose also points to this metabolic interaction. By efficiently removing both hydrogen and formate, the methanogenic archaeon improves the substrate consumption rates and pulls the glucose fermentation toward butyrate production rather than toward lactate formation. This may facilitate the butyrate-producing bacteria in a highly competitive ecosystem as the gut to successfully compete for a substrate as well as harvest extra energy from the same substrate.

We expanded our study with the interaction between *A. rhamnosivorans* and the pectin degrader B. *thetaiotaomicron* in dietary pectins (Table 3). Here, we demonstrated that the butyrogenic bacterium clearly benefits from the growth and activity of *B. thetaiotaomicron* degrading the SSPS and SBP pectin fractions. Previous studies showed that *B. thetaiotaomicron* stimulated butyrate production when colonized with *Eubacterium rectale* in germ-free mice fed with glucan (Shoaie et al., 2013) and pectin fermentation by the human microbiota also resulted in enhanced formation of butyrate (Dongowski et al., 2000). The cell density and acetate production of *B. thetaiotaomicron* indicated that this strain grew better in SSPS as compared to SBP (Table 3 and Supplementary Data S3A,B). In addition, the analysis of the monosaccharide composition revealed that more substrates were used in SSPS than that in SBP in both co-culture and monoculture (Supplementary Table S4). Nevertheless, butyrate production in the co-culture on SBP was higher than that of SSPS, indicating that *B. thetaiotaomicron* facilitated growth of *A. rhamnosivorans* in the co-cultures better in SBP as compared to SSPS. As we did not observe a significant difference in the consumption of monosaccharides in the co-culture versus monoculture, *A. rhamnosivorans* may not be able to ferment released sugars from pectin degradation by *B. thetaiotaomicron*. In addition, lactate was only detected in the monocultures but not in the co-cultures, implying that *A. rhamnosivorans* was mainly producing butyrate from lactate and acetate. The higher butyrate formation could be attributed to the increased lactate or GalA released from SBP degradation by *B. thetaiotaomicron* that was subsequently converted to butyrate by *A. rhamnosivorans*. In both mono- and co-cultures, the acetate concentration and cell numbers implied a dominating role of *B. thetaiotaomicron* (Table 3 and Supplementary Data S3A,B). Finally, the co-culture experiments illustrated a syntrophic interaction between the two species when grown with SBP and SSPS: increased butyrate levels, which may be important for gut health (Figure 3). Dietary pectin fractions have a number of proposed health-promoting benefits. A study in a rat model showed that apple pectin reduced the number of colon tumors (Ohkami et al., 1995). Orange peel pectin was shown to have a prebiotic effect that led to an increased butyrate production which was correlated with an increase in the population of *E. rectale in vitro* (Manderson et al., 2005). Crude pectin from peels of apple, carrot, lemon and orange has shown to stimulate the growth of *Bifidobacterium bifidum* and *Lactobacillus acidophilus in vitro* (Sen et al., 2014). Pectin has been shown to be potentially effective for cholesterol reduction and has anti-inflammatory and anti-carcinogenic effects (Tazawa et al., 1997; Bladergroen et al., 1999; Sherry et al., 2010). Pectin has been implicated to modulate the composition and activity of the gut microbiota in a beneficial way depending on its structural features (Larsen et al., 2019). In our study, we observed that sugar beet pectin fractions promoted butyrate production and growth of both the butyrate producing bacterium and the pectin degraders *in vitro* contributing to better understanding of another beneficial effect of SBP and SSPS on the gut bacteria.

This study revealed the occurrence of cross-feeding between anaerobes that naturally exist in the human intestinal tract and emphasized on those that are associated with butyrate formation. The butyrate-producing bacterium, *A. rhamnosivorans* benefits from the presence of specific intestinal microorganisms or substrates for its growth and butyrate production. This may be due to its high metabolic flexibility using a wide variety of mono- and disaccharide as substrates for butyrogenesis, as well as its capacity to efficiently use lactate plus acetate for butyrate production. Pectin is one of the most important sources of dietary fiber and has a number of health-promoting benefits. Our co-culture study demonstrated that these pectins promote the growth of the butyrate-producing bacteria and consequently the butyrate level via syntrophic interactions. One of the implications is that these two pectins, SPS and SSPS could be potential butyrogenic compounds to be used as prebiotic, nevertheless this needs to be further verified *in vivo* system.

Employing an *in vitro* defined co-culture system to research the microbial interaction between intestinal inhabitants of key functional groups, our study not only provides a better understanding of microbe-microbe interaction in the gut that facilitates a further buildup of a more complex microbial community but also implies potential strategic approaches to enhance intestinal butyrate formation. High butyrate production can be used as a trait to design microbe mixtures for therapeutic purposes.

## Data Availability Statement

All datasets generated for this study are included in the article/Supplementary Material.

## Author Contributions

TB, WV, and CP designed the research. TB conducted the experimental work and drafted the manuscript. HS, AS, WV, and CP contributed to the data analysis and interpretation. MJ performed the measurement on monosaccharide composition from different pectin fractions. All authors provided feedback and corrections on the manuscript, revised the intellectual content, approved the final version, and agreed to be accountable for all aspects of the work.

## Conflict of Interest

The authors declare that the research was conducted in the absence of any commercial or financial relationships that could be construed as a potential conflict of interest.
