# Supplementary material for: Mutual Metabolic Interactions in Co-cultures of the Intestinal Anaerostipes rhamnosivorans With an Acetogen, Methanogen, or Pectin-Degrader Affecting Butyrate Production
Source: Front Microbiol. 2019 Nov 1;10:2449. doi: 10.3389/fmicb.2019.02449 (PMC6839446; doi:10.3389/fmicb.2019.02449)
Supplement: Supplementary file 1 [file Table_1.DOCX]

**Monosaccharide constituent composition**

Samples taken from the monoculture of *B. thetaiotaomicron* and the co-culture in pectin after inoculation (T0) and 6 days of incubation (T6) were analyzed for their monosaccharide constituent composition using the method described elsewhere (De Ruiter, Schols, Voragen and Rombouts 1992), using duplicate aliquots of samples (25 µl for T0 and 100 µl for T6). Methanolysis was performed in 2 M HCl in dry methanol at 80^o^C for 16h, followed by hydrolysis in 2 M TFA at 121 ^o^C for 1h. The hydrolyzed samples were dried and re-dissolved in water. Aliquots of 100 ul standard solution containing 1 mg/ml each of fucose, arabinose, rhamnose, galactose, glucose, xylose, mannose, galacturonic acid and glucuronic acid were treated identical to and in parallel with the samples. The standards were diluted to 2.5, 5, 7.5 and 10 µg/ml, whereas the samples were diluted so that the concentration of monosaccharides was below 10 µg/ml.

The monosaccharides were then analyzed using a Dionex ICS5000 High Performance Anion Exchange Chromatography with Pulsed Amperometric Detection (HPAEC-PAD) system (Thermo Fisher Scientific, Waltham, MA, USA), with post-column addition equipped with a CarboPac PA-1 (2 x 250 mm) column preceded by a guard column (2 x 50 mm). The eluents used for separation were 0.1 M NaOH, 1 M sodium acetate in 0.1 M NaOH. The flow rate of the gradient pump was 0.4 ml/min. The gradient was based on a method published elsewhere (Sengkhamparn, Verhoef, Schols, Sajjaanantakul and Voragen 2009), with modifications to adapt to the different system used. First, neutral monosaccharides were eluted using a 27 min isocratic elution of water, with post-column addition of 0.1 ml/min 0.5 M NaOH.  Then, acidic monosaccharides were eluted using an 11 min gradient from 100 mM sodium acetate in 0.1M NaOH to 173 mM sodium acetate in 0.1M NaOH. After each run, the column was cleaned using 1M sodium acetate in 0.1M NaOH for 5 min, followed by 8 min isocratic elution of 0.1M NaOH to remove the acetate. The column was allowed equilibration for 15 min in the starting condition before each injection.

**References**

De Ruiter GA, Schols HA, Voragen AGJ*, et al.* 1992. Carbohydrate analysis of water-soluble uronic acid-containing polysaccharides with high-performance anion-exchange chromatography using methanolysis combined with tfa hydrolysis is superior to four other methods Analytical Biochemistry **207**: 176-185.

Sengkhamparn N, Verhoef R, Schols HA*, et al.* 2009. Characterisation of cell wall polysaccharides from okra (abelmoschus esculentus (l.) moench) Carbohydrate Research **344**: 1824-1832.
