# Supplementary material for: Mutual Metabolic Interactions in Co-cultures of the Intestinal Anaerostipes rhamnosivorans With an Acetogen, Methanogen, or Pectin-Degrader Affecting Butyrate Production
Source: Front Microbiol. 2019 Nov 1;10:2449. doi: 10.3389/fmicb.2019.02449 (PMC6839446; doi:10.3389/fmicb.2019.02449)
Supplement: Supplementary file 2 [file Table_2.DOCX]

**Supplementary data 1: Cell count in coculture of *A. rhamnosivorans* and *Blautia hydrogenotrophica*** **in lactate**. The counting was done in 106 fields. The ratio between *A. rhamnosivorans* and *Blautia hydrogenotrophica* is calculated in each field. The bold value is mean of all counted fields. The standard deviation and standard error were 0.8 and 0.08 respectively.

**
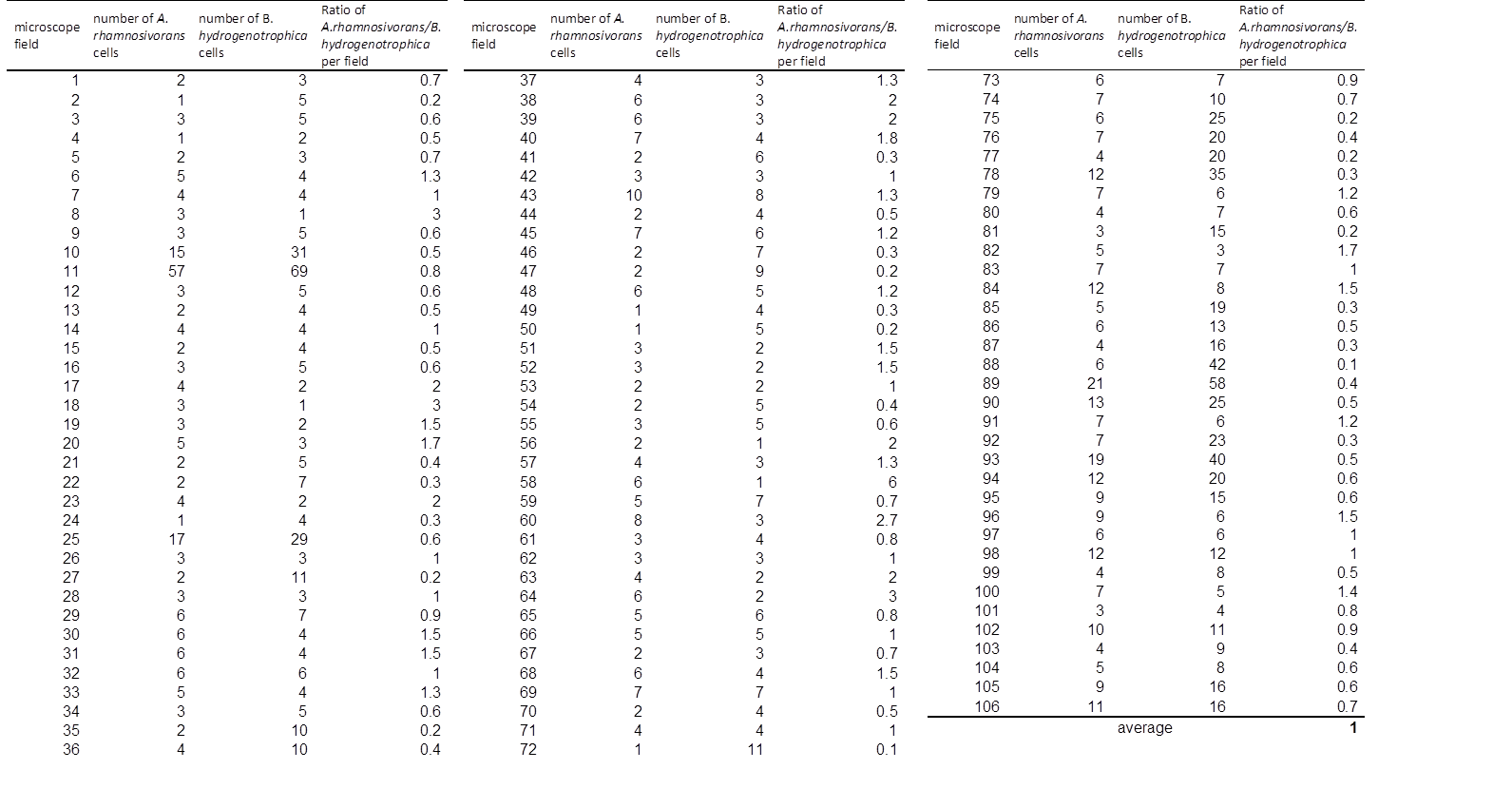
**

**Supplementary data 2: Cell count in coculture of *A. rhamnosivorans* and *M. smithii*** **in glucose**. The counting was done in 48 fields. The ratio between *A. rhamnosivorans* and *M. smithii* is calculated in each field. The bold value is mean of all counted fields. The standard deviation and standard error were 1.71 and 0.25 respectively.

**
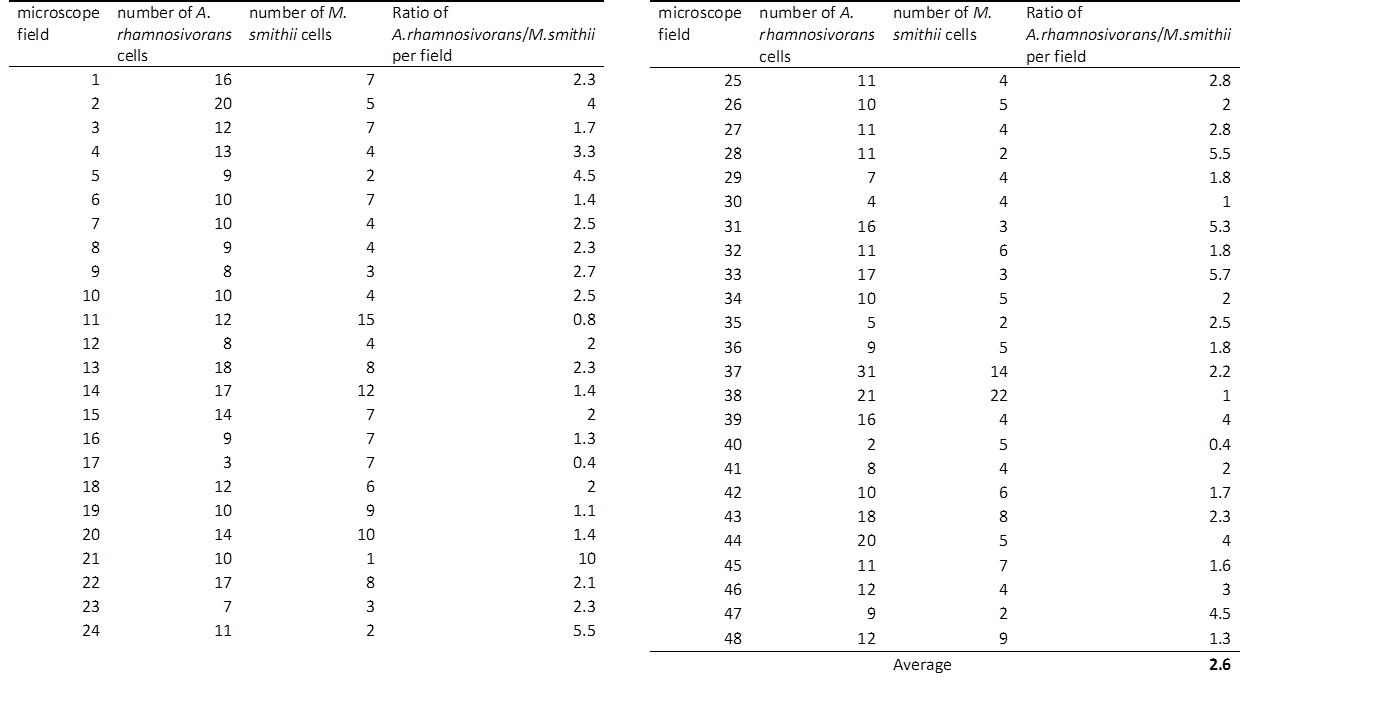
**

**Supplementary data 3-A: Cell count in coculture of *Bacteroides thetaiotaomicron* and *A. rhamnosivorans* in SBP.** The counting was done in 74 fields. The ratio between *Bacteroides thetaiotaomicron* and *A. rhamnosivorans* is calculated in each field. The bold value is mean of all counted fields. The standard deviation and standard error were 10.08 and 1.17 respectively.

**
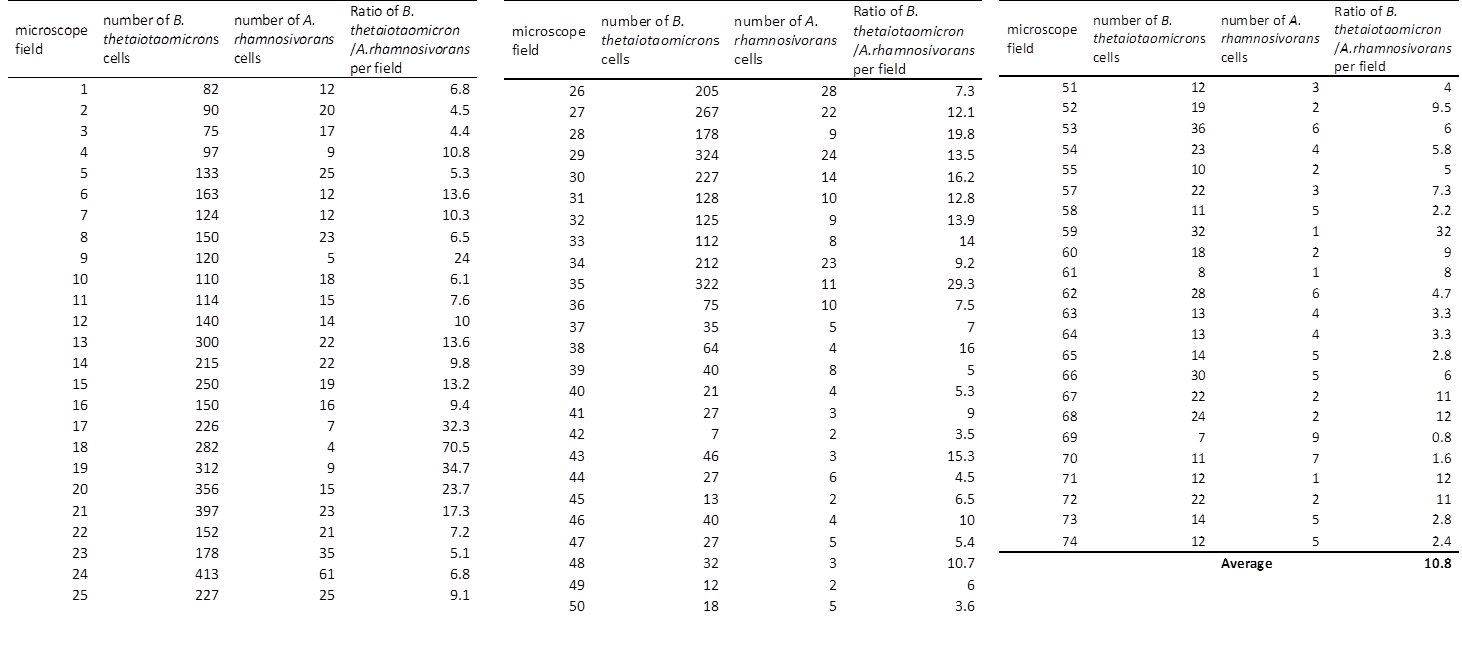
**

**Supplementary data 3-B: Cell count in coculture of *Bacteroides thetaiotaomicron* and *A. rhamnosivorans* in SSPS.** The counting was done in 84 fields. The ratio between *Bacteroides thetaiotaomicron* and *A. rhamnosivorans* is calculated in each field. The bold value is mean of all counted fields. The standard deviation and standard error were 7.41 and 0.81 respectively.

**
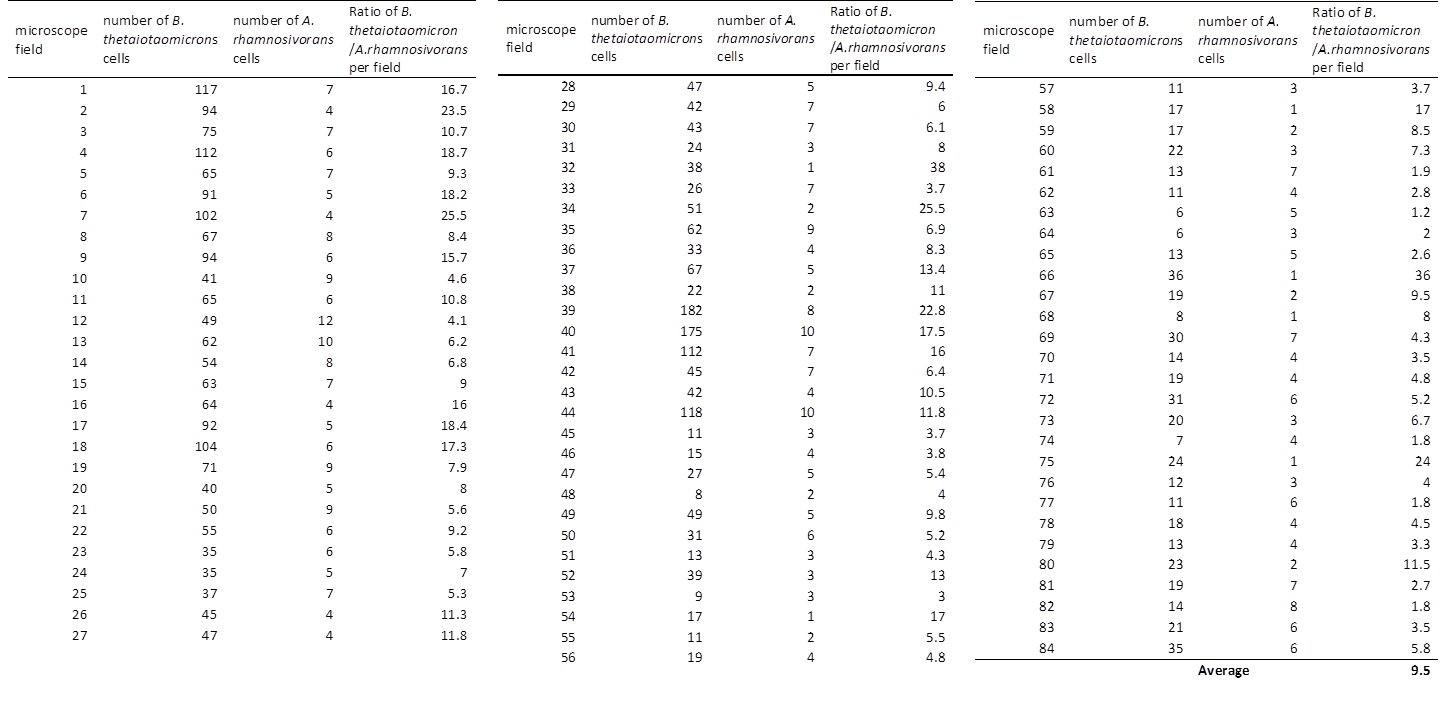
**

**Supplementary Table S1:** **Carbon and electron balance of the coculture of *A. rhamnosivorans* and *Blautia hydrogenotrophica* growing on lactate and traces of external acetate**

**
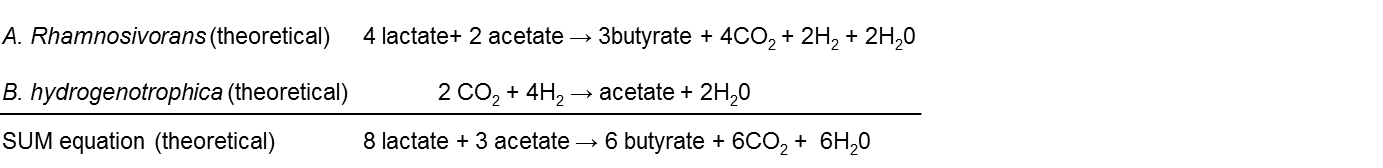
**

|  | Lactate | H_2_ | Butyrate | Acetate | CO_2_ | Sum | Recovery % | |
| --- | --- | --- | --- | --- | --- | --- | --- | --- |
| Number of Carbons/mole | 3 | 0 | 4 | 2 | 1 |  |  |  |
| Number of Electrons/mole | 12 | 2 | 20 | 8 | 0 |  |  |  |
|  |  |  |  |  |  |  |  |  |
| Consumed | 19.7 |  |  |  |  |  |  |  |
| Produced |  | 0.05 | 12.4 | 0.69 | 12.4 |  |  |  |
|  |  |  |  |  |  |  |  |  |
| ∆ Carbon | 59.1 | 0 | 49.6 | 1.38 | 12.4 | 63.38 | 107.242 |  |
| ∆ Electron | 236.4 | 0.1 | 248 | 5.52 | 0 | 253.62 | 107.2843 |  |

The carbon and electron balances were calculated after 72h of incubation. The electrons released were calculated upon the oxidation to CO_2_. The amount of formed CO_2_ is calculated based on proposed stoichiometry in the coculture. Therefore, the amount of CO_2_ is equal with the amount of butyrate.

**Supplementary Table S2:** **Carbon and electron balance of the coculture of *A. rhamnosivorans* and *Methanobrevibacter smithii* and pure culture of *A. rhamnosivorans* growing on glucose.**

|  | Substrate |  |  |  |  | Products |  |  |  |  |  |
| --- | --- | --- | --- | --- | --- | --- | --- | --- | --- | --- | --- |
|  | Glucose |  | Lactate | Acetate | Butyrate | Formate | CO_2_ | H_2_ | CH_4_ | SUM | RECOVERY % |
| Number of carbons/mole | 6 |  | 3 | 2 | 4 | 1 | 1 | 0 | 1 |  |  |
| Number of electrons/mole | 24 |  | 12 | 8 | 20 | 2 | 0 | 2 | 8 |  |  |
|  |  |  |  |  |  |  |  |  |  |  |  |
| **Co-culture** |  |  |  |  |  |  |  |  |  |  |  |
|  |  |  |  |  |  |  |  |  |  |  |  |
| Consumed | -18.15 |  |  |  |  |  |  |  |  |  |  |
| Produced |  |  | 0.33 | 6.27 | 19.73 |  | 13.31 | 1.39 | 5.06 |  |  |
|  |  |  |  |  |  |  |  |  |  |  |  |
| ∆ Carbon | -108.89 |  | 0.98 | 12.54 | 78.93 | 0.00 | 13.31 | 0.00 | 5.06 | 110.82 | -101.77 |
| ∆ Electron | -435.57 |  | 3.93 | 50.16 | 394.64 | 0.00 | 0.00 | 2.77 | 40.48 | 491.98 | -112.95 |
|  |  |  |  |  |  |  |  |  |  |  |  |
| **Pure culture** |  |  |  |  |  |  |  |  |  |  |  |
|  |  |  |  |  |  |  |  |  |  |  |  |
| Consumed | -20.13 |  |  |  |  |  |  |  |  |  |  |
| Produced |  |  | 16.06 | 4.87 | 15.23 | 20.70 | 3.5 | 2.19 |  | 62.55 |  |
|  |  |  |  |  |  |  |  |  |  |  |  |
| ∆ Carbon | -120.76 |  | 48.19 | 9.73 | 60.91 | 20.70 | 3.5 | 0.00 |  | 143.03 | -118.44 |
| ∆ Electron | -483.06 |  | 192.75 | 38.94 | 304.57 | 41.41 | 0.00 | 4.38 |  | 582.05 | -120.49 |

The carbon and electron balances were calculated after 52h of incubation. The electrons released were calculated upon the oxidation to CO_2_ . The amount of formed CO_2_ is calculated based on the assumption that 1 mol of glucose utilized releases 2 mol of CO_2_ or 1 mol formate or 2 mol lactate. Therefore CO_2_ is calculated as (2 × glucose) − formate – lactate for the pure culture and as (2 × glucose) − formate – lactate – CH_4_ for the coculture.

**Supplementary Table S3:** **Product formation of mono cultures of *B. thetaiotaomicron* and cocultures of *B. thetaiotaomicron* and *A. rhamnosivorans* growing on pectin fractions.** The experiment was performed in bicarbonate buffered medium containing 2g/l SBP; 1g/l SBP6230; 1g/l SBPOS; 0.4g/l RG-I potato; 0.2g/l RG-I apple; 1g/l SSPS as substrates. The ratio of inocula was 1:1 (2 % (v/v) of each inoculum). Product formation was quantified after 8 days of incubation. All values were corrected for time zero. ND: not detected or below detection limit. Hydrogen was not detected in any of the incubations. No growth nor product formation was observed in monocultures of *A. rhamnosivorans* in any of the pectin fractions.

| Substrates |  | Glucose  (mM) | Lactate  (mM) | Acetate  (mM) | Propionate  (mM) | Butyrate  (mM) | OD  600nm |
| --- | --- | --- | --- | --- | --- | --- | --- |
| SBP | Monoculture | ND | 1.05 | 17.64 | 2.29 | ND | 0.43 |
|  | Coculture | 0.01 | 0 | 14.96 | 2.63 | 0.74 | 0.51 |
|  |  |  |  |  |  |  |  |
| SBP 6230 | Monoculture | 0.07 | ND | 8.29 | 1.12 | ND | 0.32 |
|  | Coculture | ND | ND | 7.82 | 1.66 | ND | 0.414 |
|  |  |  |  |  |  |  |  |
| RG-I potato | Monoculture | ND | ND | 2.59 | ND | ND | 0.27 |
|  | Coculture | 0.10 | ND | 2.66 | ND | ND | 0.25 |
|  |  |  |  |  |  |  |  |
| RG-I apple | Monoculture | ND | ND | 1.21 | ND | ND | 0.15 |
|  | Coculture | ND | ND | ND | ND | ND | 0.15 |
|  |  |  |  |  |  |  |  |
| SSPS | Monoculture | ND | ND | 9.82 | ND | ND | 0.27 |
|  | Coculture | ND | ND | 11.46 | 0.82 | 0.71 | 0.33 |
|  |  |  |  |  |  |  |  |
| SBPOS | Monoculture | ND | ND | 6.80 | 0.78 | ND | 0.37 |
|  | Coculture | ND | ND | 5.75 | 0.60 | ND | 0.38 |

**Supplementary Table S4: Monosaccharide constituent composition (g/l) from monocultures of *B. thetaiotaomicron*** **and cocultures of *B. thetaiotaomicron* and *A. rhamnosivorans* and with SBP (blue) and SSPS (orange)**


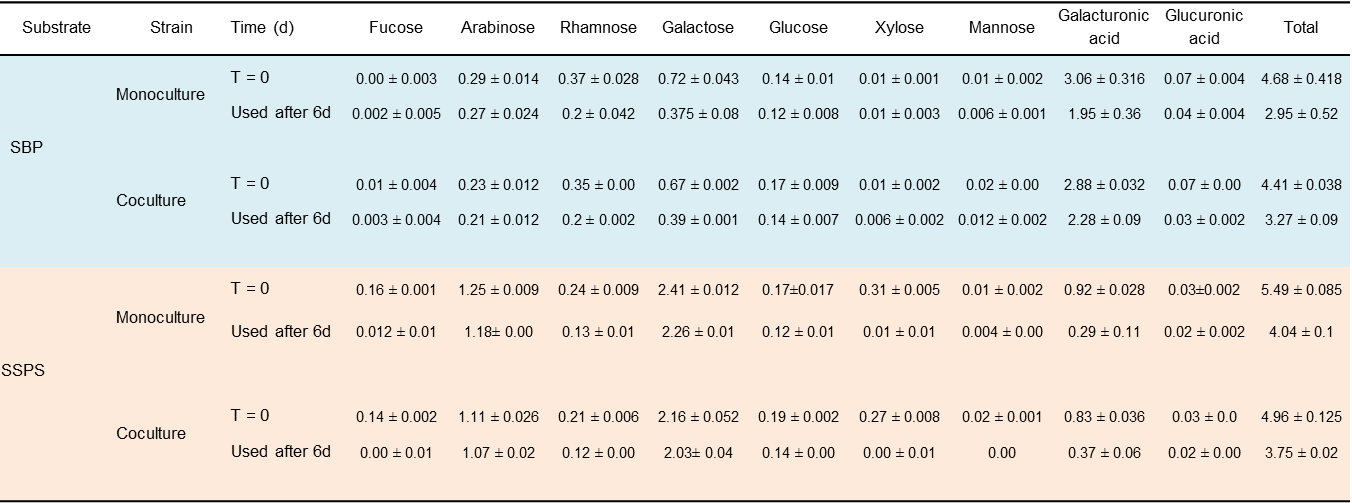


Monosaccharide constituent composition was determined directly after inoculation and after 6 days of growth. Mean and variation are shown (n=2). All experiments were done in duplicate. 4.68 g/l SBP and 5.49 g/l of SSPS was added as substrate at T0.
